# Development and worldwide use of non-lethal, and minimal population-level impact, protocols for the isolation of amphibian chytrid fungi

**DOI:** 10.1038/s41598-018-24472-2

**Published:** 2018-05-17

**Authors:** Matthew C. Fisher, Pria Ghosh, Jennifer M. G. Shelton, Kieran Bates, Lola Brookes, Claudia Wierzbicki, Gonçalo M. Rosa, Rhys A. Farrer, David M. Aanensen, Mario Alvarado-Rybak, Arnaud Bataille, Lee Berger, Susanne Böll, Jaime Bosch, Frances C. Clare, Elodie A. Courtois, Angelica Crottini, Andrew A. Cunningham, Thomas M. Doherty-Bone, Fikirte Gebresenbet, David J. Gower, Jacob Höglund, Timothy Y. James, Thomas S. Jenkinson, Tiffany A. Kosch, Carolina Lambertini, Anssi Laurila, Chun-Fu Lin, Adeline Loyau, An Martel, Sara Meurling, Claude Miaud, Pete Minting, Serge Ndriantsoa, Simon J. O’Hanlon, Frank Pasmans, Tsanta Rakotonanahary, Falitiana C. E. Rabemananjara, Luisa P. Ribeiro, Dirk S. Schmeller, Benedikt R. Schmidt, Lee Skerratt, Freya Smith, Claudio Soto-Azat, Giulia Tessa, Luís Felipe Toledo, Andrés Valenzuela-Sánchez, Ruhan Verster, Judit Vörös, Bruce Waldman, Rebecca J. Webb, Che Weldon, Emma Wombwell, Kelly R. Zamudio, Joyce E. Longcore, Trenton W. J. Garner

**Affiliations:** 1 Department of Infectious Disease Epidemiology, School of Public Health, Faculty of Medicine (St Mary’s campus), Imperial College London, London, W2 1PG UK; 2 Centro de Investigación para la Sustentabilidad, Facultad de Ecología y Recursos Naturales, Universidad Andres Bello, Republica 440, Santiago, Chile; 3 Laboratory of Behavioral and Population Ecology, School of Biological Sciences, Seoul National University, Seoul, 08826 South Korea; 4 CIRAD, UMR ASTRE, F-34398 Montpellier, France; ASTRE, Univ Montpellier, CIRAD, INRA, Montpellier, France; 5 One Health Research Group, College of Public Health, Medical and Veterinary Sciences, James Cook University, Townsville, Queensland 4811 Australia; 6 Agency for Population Ecology and Nature Conservancy, Gerbrunn, Germany; 7 Museo Nacional de Ciencias Naturales, CSIC c/Jose Gutierrez Abascal 2, 28006 Madrid, Spain; 8 Laboratoire Ecologie, évolution, interactions des systèmes amazoniens (LEEISA), Université de Guyane, CNRS, IFREMER, 97300 Cayenne, French Guiana; 9 CIBIO - Centro de Investigação em Biodiversidade e Recursos Genéticos, InBIO, Universidade do Porto, 4485-661 Vairão, Portugal; 10 Institute of Zoology, Regent’s Park, London, NW1 4RY UK; 11 Conservation Programmes, Royal Zoological Society of Scotland, Edinburgh, UK; 12 Department of Integrative Biology, Oklahoma State University, 113 Life Sciences West, Stillwater, OK 74078 USA; 13 Life Sciences, The Natural History Museum, London, SW7 5BD UK; 14 Department of Ecology and Genetics, EBC, Uppsala University, Norbyv. 18D, SE-75236 Uppsala, Sweden; 15 Zoology Division, Endemic Species Research Institute, 1 Ming-shen East Road, Jiji, Nantou, 552 Taiwan; 16 Helmholtz Centre for Environmental Research – UFZ, Department of Conservation Biology, Permoserstrasse 15, 04318 Leipzig, Germany; 17 ECOLAB, Université de Toulouse, CNRS, INPT, UPS, Toulouse, France; 18 Department of Pathology, Bacteriology and Avian Diseases, Faculty of Veterinary Medicine, Ghent University, Salisburylaan 133, B-9820 Merelbeke, Belgium; 19 PSL Research University, CEFE UMR 5175, CNRS, Université de Montpellier, Université Paul-Valéry Montpellier, EPHE, Biogéographie et Ecologie des vertébrés, Montpellier, France; 20 Amphibian and Reptile Conservation (ARC) Trust, 655A Christchurch Road, Boscombe, Bournemouth, Dorset, BH1 4AP UK; 21 Durrell Wildlife Conservation Trust, Madagascar Programme, Antananarivo, Madagascar; 22 Department of Evolutionary Biology and Environmental Studies, University of Zurich, Winterthurerstrasse 190, 8057 Zurich, Switzerland; 23 National Wildlife Management Centre, APHA, Woodchester Park, Gloucestershire, GL10 3UJ UK; 24 Non-profit Association Zirichiltaggi - Sardinia Wildlife Conservation, Strada Vicinale Filigheddu 62/C, I-07100 Sassari, Italy; 25 ONG Ranita de Darwin, Nataniel Cox 152, Santiago, Chile; 26 Unit for Environmental Sciences and Management, Private Bag x6001, North-West University, Potchefstroom, 2520 South Africa; 27 Collection of Amphibians and Reptiles, Department of Zoology, Hungarian Natural History Museum, Budapest, Baross u 13., 1088 Hungary; 28 School of Biology and Ecology, University of Maine, Orono, Maine, 04469 USA; 29 Department of Ecology and Evolutionary Biology, University of Michigan, Ann Arbor, Michigan 48109 USA; 30 Laboratório de História Natural de Anfíbios Brasileiros (LaHNAB), Departamento de Biologia Animal, Instituto de Biologia, Universidade Estadual de Campinas, Campinas, São Paulo, 13083-862 Brazil; 31 Department of Ecology and Evolutionary Biology, Cornell University, Ithaca, New York, 14853 USA; 32 Centre for Ecology, Evolution and Environmental Changes (CE3C), Faculdade de Ciências da Universidade de Lisboa, Lisboa, Portugal; 33 IUCN SSC Amphibian Specialist Group-Madagascar, 101, Antananarivo, Madagascar; 34 Info Fauna Karch, Université de Neuchâtel, Bellevaux 51, UniMail Bâtiment 6, 2000 Neuchâtel, Switzerland; 35 Centre for Genomic Pathogen Surveillance, Wellcome Genome Campus, Cambridgeshire, UK

## Abstract

Parasitic chytrid fungi have emerged as a significant threat to amphibian species worldwide, necessitating the development of techniques to isolate these pathogens into culture for research purposes. However, early methods of isolating chytrids from their hosts relied on killing amphibians. We modified a pre-existing protocol for isolating chytrids from infected animals to use toe clips and biopsies from toe webbing rather than euthanizing hosts, and distributed the protocol to researchers as part of the BiodivERsA project *RACE*; here called the *RML* protocol. In tandem, we developed a lethal procedure for isolating chytrids from tadpole mouthparts. Reviewing a database of use a decade after their inception, we find that these methods have been applied across 5 continents, 23 countries and in 62 amphibian species. Isolation of chytrids by the non-lethal *RML* protocol occured in 18% of attempts with 207 fungal isolates and three species of chytrid being recovered. Isolation of chytrids from tadpoles occured in 43% of attempts with 334 fungal isolates of one species (*Batrachochytrium dendrobatidis*) being recovered. Together, these methods have resulted in a significant reduction and refinement of our use of threatened amphibian species and have improved our ability to work with this group of emerging pathogens.

## Introduction

A major consequence of globalisation has been the increase of invasive species owing to trade in live animals and plants. A further outcome of this process is the concomitant rise of novel emerging fungal pathogens (EFPs^1^) as these infections are moved within trade networks and establish in uninfected regions – an example of fungal ‘pathogen pollution’^2^. Whilst EFPs can affect humans, they have also been broadly detrimental to natural populations of plants and animals, leading to worldwide losses of biodiversity. This dynamic has been most apparent across amphibians, where EFPs leading to population extirpation and species extinctions have contributed to amphibians now being the most endangered class of vertebrate^3,4^. In particular, emergence of parasitic fungi in the genus *Batrachochytrium* (phylum Chytridiomycota, order Rhizophydiales) have played a major role in driving amphibian population and species declines worldwide^5,6^.

While a single species, *Batrachochytrium dendrobatidis* (*Bd*), was originally thought to have caused the ongoing panzootic^7^, we now know that amphibian chytridiomycosis is caused by a much broader swathe of phylogenetic diversity than was previously thought^8,9^. Next-generation sequencing and phylogenomic analyses have shown that *Bd sensu stricto* is composed of deep genetic lineages which are emerging through international trade in amphibians^10–12^. Superimposed upon this background of trade-associated lineages of *Bd* has come the recent discovery of a new species of pathogenic chytrid, also within the Rhizophydiales, *B. salamandrivorans*^13^. This pathogen has rapidly extirpated European fire salamanders (*Salamandra salamandra*) in the Netherlands and a broad screening of urodeles has shown that *Bsal* occurs naturally in southeast Asia where it appears to asymptomatically infect salamander and newt species^14^.

The ability to isolate and culture both *Bd* and *Bsal* has played a key role in their discovery and by catalysing research into their pathogenesis and virulence^15–17^, phenotypic characteristics^18–20^ and a wealth of experimental studies on epidemiologically relevant parameters^21–23^. Longcore *et al*.^24^ first isolated *Bd* from infected amphibians by modifying techniques used to isolate other chytrids^25^. Longcore cleaned small (<0.5 mm dia) pieces of *Bd*-infected leg and foot skin by wiping them through agar and then placed skin pieces onto a clean plate of nutrient agar containing penicillin G and streptomycin. This method worked well for isolating from dead animals sent by courier from North and Central America. The method, however, requires euthanizing potentially healthy animals if their infection status was unknown. Further, it is difficult to perform these techniques in remote regions that lack suitable laboratory facilities, and the lethal sampling of amphibians may be contraindicated if the species is endangered, protected or located in protected areas.

We confronted this issue in a 2008–2014 project funded by BiodivERsA (http://www.biodiversa.org) – *RACE*: Risk Assessment of Chytridiomycosis to European amphibian biodiversity^26^. One of the objectives of this project was to adjust the original protocol of Longcore *et al*.^24^ to (i) reduce the need to kill adult amphibians, (ii) improve rates of chytrid isolation by allowing the use of more animals, (iii) develop protocols that enabled isolation in a field setting, and, (iv) integrate the data into the GPS-smartphone enabled epidemiological software application *Epicollect*^27,28^. Furthermore, ‘forewarned is forearmed’ and we wished to determine whether the protocol was able to isolate other species of chytrid that are members of the amphibian skin microbiota, and that may present a biosecurity risk. This need to more broadly characterise global chytrid biodiversity was met by using resources from *RACE* to train researchers worldwide in chytrid isolation techniques to provide opportunities to characterise novel chytrids as they were discovered.

In addition to the non-lethal isolation protocol, a lethal method was developed in parallel to isolate chytrids from the mouthparts of larval amphibians. We describe this method as a refinement to the main isolation protocol.

## Methods

### Non-lethal field isolation of chytrids

Animals were captured and held in separate plastic bags or suitable containers until ready for processing (Supp. Info. *RML* Protocol 1 and Supp. Info. Swabbing Protocol 2). Using clean gloves and sterilized dissection scissors or scalpel blades, the terminal 1–2 mm of the phalanges of the 4^th^ hind toe (counting from the proximal toe) was clipped and laid onto the surface of an mTGhL + antibiotic (200 mg/L penicillin-G and 400 mg/L streptomycin sulphate) agar plate. Alternatively, ~1 mm toe-webbing biopsy punches were taken (Sklar instruments, PA, USA) then laid on a plate. This allowed multiple animals to be processed rapidly in the field. Subsequently, each tissue sample was transferred to a second plate with a sterile needle or forceps then cleaned (as far as possible) of surface-contaminating bacteria and fungi by dragging it through the agar-medium. The needle or forceps was then used to place the tissue sample into a sterile 2 ml screw-cap microtube containing liquid mTGhL medium with antibiotics (200 mg/L penicillin-G and 400 mg/L streptomycin sulphate), then stored in a cool, dry place. While 4 °C appears optimal, we have successfully used shaded regions of streams to cool cultures when refrigeration was not immediately available and have even held tubes and plates for several days at >10 °C until suitable storage conditions were available.

Once back in the laboratory, samples in tubes were visually screened for evidence of yeast or bacterial contamination (when the media takes on a ‘cloudy’ appearance), or mycelial ‘balls’ around the toe that are evidence of non-chytrid fungal contaminants. Visibly clear samples were decanted into a single well of a sterile 12-well lidded culture plate then incubated at 18 °C for up to 4 weeks, topping up with extra medium to counter evaporation as necessary. Depending on the size of the initial tissue sample, toe clips and webbing were divided into several smaller samples before transferring to liquid culture media.

### Isolating chytrids from tadpoles

**Figure 1:**
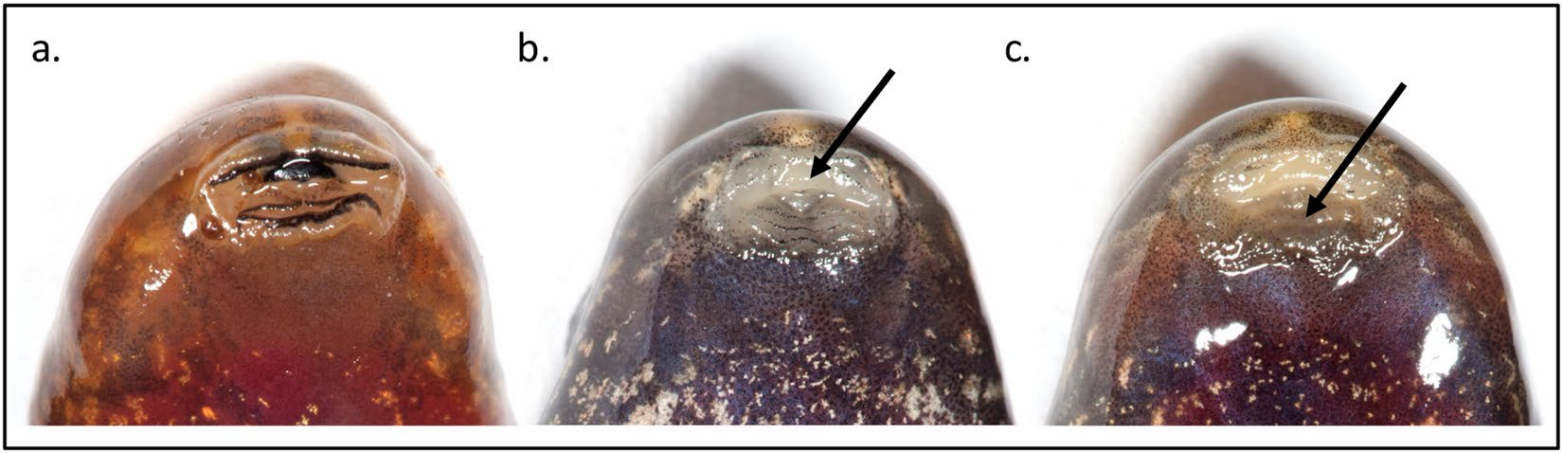
Oral deformities in tadpoles of *Hylodes phyllodes* caused by *Batrachochytrium dendrobatidis* infection. (**a**) healthy tadpole mouthparts, (**b**) depigmentation of jaw sheath and (**c**) depigmentation of tooth-rows as a consequence of infection.

Tadpoles often have higher burdens of infection than adults, especially long-lived tadpoles^29^, and have higher densities and encounter rates than adults. As incomplete data exists as to which amphibian species raise tadpoles that are susceptible to *Bd*, in practice tadpoles from a range of species should be tested. Where tadpoles are large and infections heavy, tadpoles were microscopically prescreened with a dissecting microscope or hand lens in order to detect areas of depigmentation and hyperkeratosis within mouth parts, especially the jaw sheaths, that are associated with infection^30–33^ (Fig. 1). Tadpoles are euthanized using a humane method that does not affect the fungi (e.g., overdose of MS-222) before excising their mouthparts and these preliminary microscopic screens enabled us to use only a small number of animals to isolate chytrids. Additionally, uninfected and naïve tadpoles that were reared in captivity were used as live substrates to bait chytrids from adult amphibians with low levels of *Bd* infection^34^.

When using tadpoles to bait *Bd* from infected adult amphibians, tadpoles from a susceptible species such as *Bombina orientalis*^34^ were co-housed with an infected animal. Susceptible tadpoles were reared until gills were resorbed and animals were free-swimming and feeding (developmental Gosner stage 25), because at earlier stages they are still developing the keratinized mouthparts. Each tadpole container was then immersed within a similar but larger container that held at least one chytrid-infected animal. Water exchange between the infected and bait animal containers occurred through small holes (<0.3 mm) drilled into the bottom of the walls of the smaller internal containers. Animals were held in these conditions for between 2 and 4 weeks at species-appropriate conditions. Tadpoles were periodically examined every fourth day for the presence of the depigmented areas in the jaw sheaths that have been associated with chytrid infection.

Isolating chytrids from tadpoles first required killing by immersion in a 5 g/L solution of MS-222^35^ or other approved method. Note that anaesthetics that contain ethanol, such as phenoxyethanol^36^, should be avoided as these kill chytrids while MS222 is not toxic^37^. We then dissected out keratinized jaw sheaths and cleaned the entire sheath, or sections, as above using an agar plate with antibiotics (^24^; Supp. Info. *RML* Protocol 1). Cleaned sections were then placed singly into sterile 12-well culture plates with 1 mL liquid media + antibiotics, or onto agar plates with 6–10 sections per plate, and incubated at 10–20 °C.

Because zoospore release may occur immediately, especially from tadpole mouthparts, cultures were examined with an inverted microscope for the presence of active zoospores every day for up to one week following the day that they were initiated. After that, checks every two days were sufficient.

### Culture and diagnosis of chytrid isolates

Subsequent culture methods for *Bd* followed those of Longcore *et al*.^24^. When isolation of *Bsal* was anticipated an incubation temperature of 15 °C was required^38^ whereas a temperature of 18–22 °C is closer to the measured growth optimum of *Bd*^23,24^. Once growth of zoospores and/or zoosporangia was observed, 100–500 µL volume of the culture was transferred by pipette to a new 12-well plate with liquid medium and no antibiotics, and incubated at 15–20 °C. All successfully cultured isolates were subcultured into larger volumes, then centrifuged at 1700 rpm for 10 min before cryopreservation. A portion of the initial pellet was also be used for DNA extraction, while the remaining volume was resuspended in 10% dimethyl sulfoxide (DMSO) and 10% fetal calf serum (FCS) in liquid media and transferred into six 2 mL cryotubes for cryopreservation at −80 °C^39^.

We confirmed the identity of *Bd* and *Bsal* by quantitative PCR with an MGB Taqman probe assay in either single-plex or multiplex^40,41^. We identified non-*Batrachochytrium* chytrids by sequencing appropriate regions of the ribosomal RNA gene with universal fungal primers followed by comparison against OTUs held in UNITE database (Unified system for DNA-based fungal species linked to classification: https://unite.ut.ee) to establish a species-hypothesis for the chytrid isolate in question^42^. If further genetic data were required, then multilocus analysis or whole-genome sequencing was undertaken using chytrid-specific methods^1,7,16,43^.

### Collation of data

To track and report chytrid isolation for the *RACE* project, we used a generic data collection tool that allows the collection and submission of geotagged data forms from field locations, *Epicollect5* (https://five.epicollect.net). This software has the advantage that it can be used on mobile devices with or without internet connection, and allows the immediate sharing of data across the research community. Our database at https://five.epicollect.net/project/bd-global-isolation-protocol included the following data fields: Date; Continent, Country, Site name; Latitude/Longitude; Wild caught or trade?; Amphibian species; Life history stage; Number sampled; Chytrid isolated?; Number isolated; Species of chytrid isolated; Chytrid lineage; Photograph of amphibian; Name of researchers.

All field-collection and application of protocols were performed in accordance with the relevant local guidelines, regulations and licensing. Experimental protocols were approved after ethical review by the Imperial College and Institute of Zoology ethical review committees and were performed under UK Home Office Project Licences held by MC Fisher and TWJ Garner.

### Data accessibility

https://five.epicollect.net/project/bd-global-isolation-protocol.

## Results

The ‘*RACE* modified Longcore (*RML*) Protocol’ for the non-lethal isolation of chytrids from amphibians is detailed in Supp. Info. 1. Researchers should ensure that they have the relevant licences, permits and permissions from ethical committees to follow the *RML* protocol 1, swabbing protocol 2 and isolation from larval amphibians.

**Figure 2:**
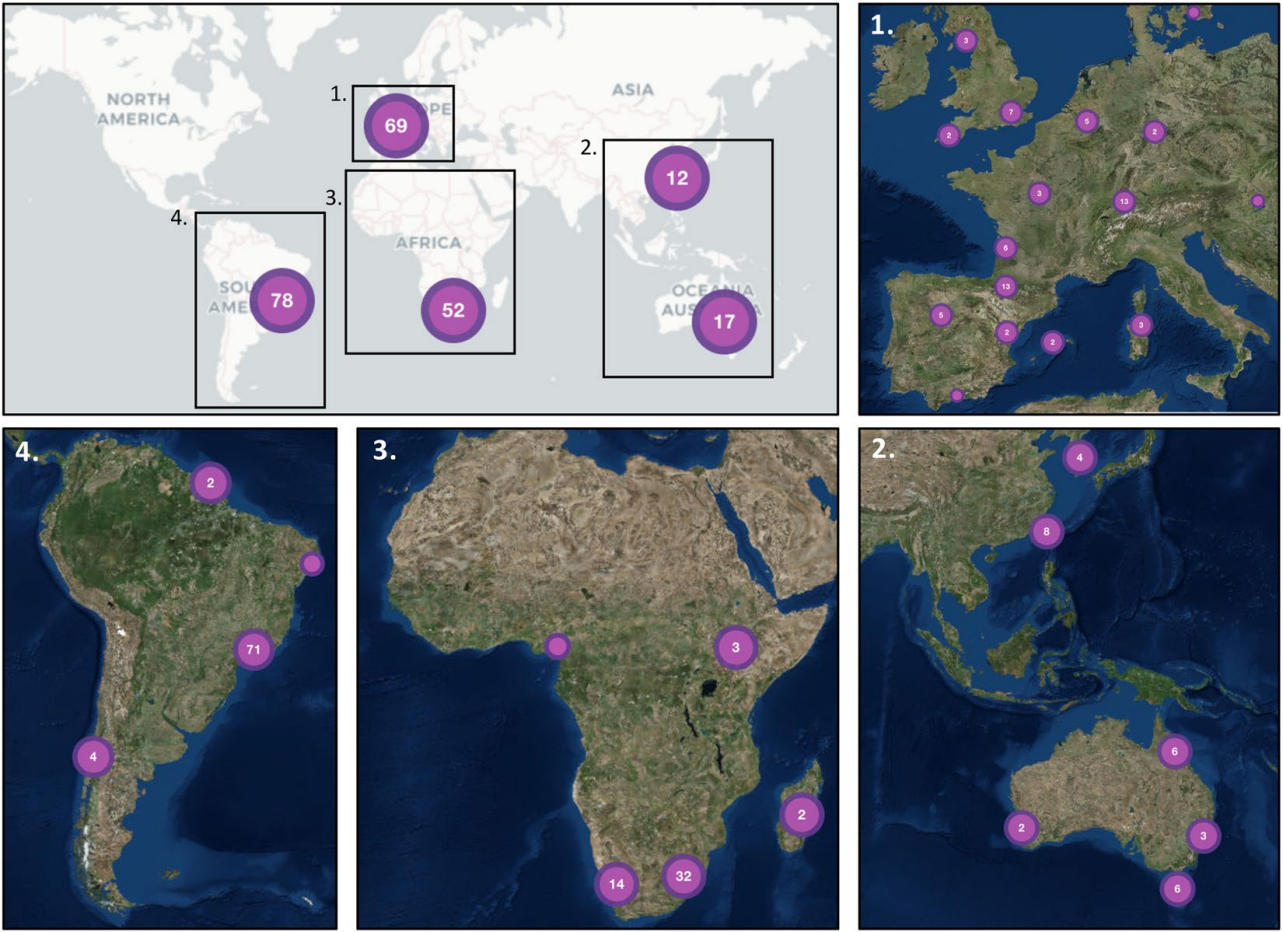
Worldwide distribution of sites where the RML Longcore protocol has been used to isolate chytrids. Numbers denote the quantity of amphibian species investigated. A browseable version of this Epicollect 5 map can be accessed at https://five.epicollect.net/project/bd-global-isolation-protocol. Tiles © Esri — Source: Esri, i-cubed, USDA, USGS, AEX, GeoEye, Getmapping, Aerogrid, IGN, IGP, UPR-EGP, and the GIS User Community and OpenStreetMap © OpenStreetMap.org contributors licence CC BY-SA (https://creativecommons.org/licenses/by-sa/2.0/).

Following the formalisation and distribution of the *RACE* protocols, our Epicollect5 project summarised chytrid surveys from 2007 through to 2017 (Table 1). The Epicollect5 database can be spatially visualised at https://five.epicollect.net/project/bd-global-isolation-protocol/data. Figure 2 depicts the isolation of amphibian-associated chytrids using the *RACE* protocols from 5 continents (Africa, Asia, Australia, Europe and South America), 23 countries, 239 sampling episodes, and from latitudes spanning −44.1 S (*Batrachyla antartandica*, Chile) through to 55.6 N (*Bufo viridis*, Sweden). Chytrids have been non-lethally isolated from 1,906 animals comprising 34 amphibian species, of which 28 were anuran and 5 were caudatan species. Of the *Bd* isolated, 170 (80%) were determined to be *Bd*GPL, 5 (2%) were *Bd*CAPE, 34 (16%) were *Bd*BRAZIL, 1 (>1%) was *Bd*CH and 3 (1%) were hybrids. The database also contains 5 records of chytrids that were non-lethally sampled from the amphibian trade.

**Table 1:** Non-lethal isolation of chytrids from adult and juvenile amphibians.

Continent	Country	*n* Species^a^	*n* Sampled^b^	*n* Chytrid^c^	Chytrid species
Africa	Madagascar	2	145	2	*Kappamyces* sp.
	Cameroon	1	30	1	*B. dendrobatidis*
	Ethiopia	1	5	1	*B. dendrobatidis*
	South Africa	6	179	45	*B. dendrobatidis*
Asia	South Korea	2	28	10	*B. dendrobatidis*
	Taiwan	3	103	13	*B. dendrobatidis/Kappamyces* sp.
Australia	Australia	1	2	2	*B. dendrobatidis*
Europe	Belgium	1	11	2	*B. dendrobatidis*
	France	2	261	70	*B. dendrobatidis*
	Hungary	1	15	3	*B. dendrobatidis*
	Italy	1	14	4	*B. dendrobatidis*
	Portugal	1	5	1	*Rhizophydium* sp.
	Spain	4	198	37	*B. dendrobatidis*
	Sweden	1	23	5	*B. dendrobatidis*
	Switzerland	1	30	1	*B. dendrobatidis*
	UK	4	50	8	*B. dendrobatidis*
South America	Chile	1	10	1	*B. dendrobatidis*
	French Guiana	2	66	2	*B. dendrobatidis*
Trade	n/a	4	15	5	*B. dendrobatidis*

^a^Number of amphibian species sampled, ^b^total numbers of amphibians sampled, ^c^number of chytrids isolated.

### Non-lethal isolation from adult and juvenile amphibians

In total, 1,152 animals were non-lethally sampled, recovering 207 chytrid isolates and resulting in a recovery rate of 18% (~1 isolate per 5 animals sampled). Of these chytrids, 203 (98%) were *Bd*, 2 were *Rhizophydium* sp., 2 were *Kappamyces* sp. and none were *Bsal* (Table 1). Of the *Bd* isolated, 42 (88%) were determined to be *Bd*GPL, 5 (10%) were *Bd*CAPE, and 1 (2%) was *Bd*CH.

### Isolation of chytrids from larval amphibians

In total, 784 tadpoles were sampled recovering 334 chytrid isolates and resulting in a recovery rate of 43% (~1 isolate per 2–3 animals sampled) (Table 2). Isolates were recovered from 34 species of amphibian, all of which were anurans. These chytrid isolates were all *Bd* and, of the lineages recorded, 128 (78%) were *Bd*GPL, 34 (20%) were *Bd*BRAZIL and 3 (2%) were hybrids.

**Table 2:** Isolation of *Batrachochytrium dendrobatidis* from mouthparts of larval amphibians.

Continent	Country	Host species	Larvae sampled	*Bd* isolates
Africa	Ethiopia	1	36	1
	Uganda	1	20	1
	South Africa	2	88	11
Asia	Taiwan	1	15	1
Australia	Australia	8	54	33
Europe	Belgium	2	2	2
	Netherlands	1	1	1
	France	1	138	38
	Germany	1	10	4
	Spain	3	19	7
	Switzerland	1	42	15
South America	Chile	2	28	4
	Brazil	17	353	217

Baiting chytrid isolates from live adult animals using tadpoles was used successfully in South Korean *Bombina orientalis* as previously described^34^. Here, six tadpoles were co-housed with adult *B. orientalis*, yielding a single isolate of *Bd* for each attempt equating to a rate of success of ~20%.

## Discussion

The *RML* protocol, based on the original suggestions of Joyce Longcore for the non-lethal isolation of chytrids from amphibians, has been a success with isolates of chytrids recorded from five continents. There are likely many other unrecorded uses of this method because this protocol has been widely dispersed during the 5-year span (2008–2014) of the *RACE* project which trained a cohort of amphibian disease researchers in these techniques.

In some circumstances chytrids could not be recovered from toe-clips when sampling populations with persistent infection despite repeated attempts. This was particularly evident when the prevalence and burden of chytrid infections in surveys was low^14,34,44^ or when host species occupied habitats with high bacterial, non-target fungal contaminants, or both. In these situations we isolated chytrids from tadpole mouthparts as an associated method to the *RML* protocol. The value of the *RML* protocol in propelling forward research on amphibian chytridiomycosis has been very clear: for instance, of the 59 scientific papers produced by *RACE*, 15 directly used isolates of *Bd* that were generated by this protocol for experimental trials. Further, subsequently many more studies using these isolates have extended our knowledge of the genetic diversity of *Bd*^7,8,43,45^, the development of novel diagnostics^46^, the genetic repertoire that underpins the virulence of these pathogens^16,17^ and the biogeographic distributions of *Bd* diversity worldwide^8,45^.

Clearly some uncontrolled biases and unanswered questions in these studies need attention. First, the majority of *Bd* isolates belong to the *Bd*GPL lineage. This could be because this lineage is more widespread (and therefore more readily recovered) than other lineages^47^. Alternatively, the intensity of *Bd*GPL infections or its rate of zoospore production may be higher than for other lineages, which would also equate to a higher rate of isolation. To achieve a true and unbiased understanding of the distribution of these lineages, a lineage-specific diagnostic will need to be developed and deployed. Second, if lineage-specific differences in the probability of successful isolation exist, then mixed infections where these lineages co-occur may not be detected. This can be controlled for by isolating and genotyping many isolates from a single host and population, although this may not fully account for this bias. A related bias is that not all infectious species of chytrid will respond equally to culturing attempts. For instance, despite known attempts to isolate *Bsal* from across its endemic southeast Asian range using the protocol, to date no successful isolations of *Bsal* have been recorded. This is likely due to a combination of the low prevalence and burden of infection in salamanders and newts combined with the low initial growth-rate of *Bsal*^13,14^. With the *RML* protocol, however, workers have been able to isolate non-*Bd* species of chytrid (*e.g., Kappamyces* spp. and *Rhizophydium* sp. Table 1). This diversity likely represents only a fraction of the diversity of amphibian-associated chytrids that occur, and non-biased estimators of this diversity by, for instance, profiling the nuclear ribosomal RNA cistron^42^, are sorely needed.

In this age of the global amphibian crisis, research on the affects of chytrid infections is transitioning to attempts to mitigate their impacts^48–50^. Both of these research streams benefit from the availability of chytrid isolates, but the ethics behind these research programs can be improved. To that end, our data on isolation success suggest that tadpoles are a better target for isolation than metamorphosed animals. This is to some degree unfortunate, because isolation from tadpoles requires killing. However, we have outlined one refinement where captive reared tadpoles can be used to ‘bait’ infections from wild-caught amphibians to isolate chytrids without killing adult amphibians. Here, it is important to recognise that amphibians that have been co-housed in collections should not be returned to the wild owing to the danger of cross-transmission of pathogens during husbandry^51^. If it is necessary to isolate chytrids directly from wild tadpoles without using bait animals, we suggest that researchers focus on more fecund species with long larval periods as the focal species in aquatic amphibian communities. Removal of small numbers of tadpoles when clutch sizes are in the hundreds or thousands should ensure minimal ecological impact; for this reason sacrificing tadpoles is preferable to killing adult animals.

The extent to which toe-clipping affects the fitness of amphibians has been much debated^52,53^). Toe-clipping has been shown to decrease amphibian survival, but this effect, when present, is linearly related to the number of toes removed^54,55^. For the single toe-clip that the *RML* protocol requires, reduction in survival appears to be negligible^53,56^, and toe clipping is certainly preferred to killing the animal. Attention should be paid to this issue, however, and, where appropriate, survival estimates should be undertaken to determine the health implications of this procedure. Also, antiseptic and analgesic protocols can be considered to ensure that wounds where tissue samples are excised are at low risk of secondary infection^57^.

In summary, modification of Longcore’s original *Bd*-isolation protocol^24^ has enabled a broad community of scientists to engage with research on emerging chytrid pathogens of amphibians. This research has had an impact worldwide, and is contributing to the ongoing dialogue that is occurring among scientists, conservationists and policy-makers about how we might mitigate against these infections now and into the future^58^.

## Electronic supplementary material

RML protocol

Swabbing protocol
