# Supplementary material for: Development and worldwide use of non-lethal, and minimal population-level impact, protocols for the isolation of amphibian chytrid fungi
Source: Sci Rep. 2018 May 17;8:7772. doi: 10.1038/s41598-018-24472-2 (PMC5958081; doi:10.1038/s41598-018-24472-2)
Supplement: Supplementary file 1 — RML protocol [file 41598_2018_24472_MOESM1_ESM.pdf]

RACE **Modified Longcore (*RML*) protocol to isolate chytrid fungi from amphibians**

Manuscript: Development and worldwide use of non-lethal, and minimal population-level impact, protocols for the isolation of amphibian chytrid fungi

Matthew C. Fisher, Pria Ghosh, Jennifer M. G. Shelton, Kieran Bates, Lola Brookes, Claudia Wierzbicki, Gonçalo M. Rosa, Rhys A. Farrer, David M. Aanensen, Mario Alvarado-Rybak, Arnaud Bataille, Lee Berger, Susanne Böll, Jaime Bosch, Frances C. Clare, Elodie Courtois, Angelica Crottini, Andrew A. Cunningham, Thomas M. Doherty-Bone, Fikirte Gebresenbet, David J. Gower, Jacob Höglund, Timothy Y. James, Thomas S. Jenkinson, Tiffany A. Kosch, Carolina Lambertini, Anssi Laurila, Chun-Fu Lin, Adeline Loyau, An Martel, Sara Meurling, Claude Miaud, Pete Minting, Serge Ndriantsoa, Frank Pasmans, Tsanta Rakotonanahary, Falitiana C. E. Rabemananjara, Luisa P. Ribeiro, Dirk S. Schmeller, Benedikt R. Schmidt, Lee Skerratt, Freya Smith, Claudio Soto-Azat, Giulia Tessa, Luís Felipe Toledo, Andrés Valenzuela-Sánchez, Ruhan Verster, Judit Vörös, Bruce Waldman, Rebecca J. Webb, Che Weldon, Emma Wombwell, Kelly R. Zamudio, Joyce E. Longcore, Trenton W. J. Garner

First make sure that you have all the local permits and permissions from ethical committees to undertake the *RML* protocol

Equipment

- Dissection scissors or fine fingernail scissors or scalpel blades
- Needles or fine forceps
- 70% ethanol or isopropanol to clean needles and dissection scissors between animals
- Gloves
- Liquid medium – either 1% tryptone (1%T) or mTGhL – with and without penicillin and streptomycin[1]
- 1.5 or 2ml sterile eppendorf or centrifuge tubes filled with 1ml of liquid nutrient medium. Fill some tubes with medium containing antibiotics, some without. Number the tubes
- Nutrient agar plates + penicillin and streptomycin
- Screw-topped, glass, reusable culture containers containing sterilized liquid nutrient medium. Alternatively, plastic, sterile, 25 ml tissue-culture flasks

Procedure

1. Captured amphibians should be held individually in plastic bags in appropriate conditions until ready for processing.
2. Wearing gloves, hold amphibian firmly at the top of its back legs.

[1] Media for isolation and growth of *Batrachochytrium* species can be either mTGhL as used in Longcore *et al* 1999 and in the *RACE* protocol or 1% tryptone as used by Longcore (https://umaine.edu/chytrids/batrachochytrium-dendrobatidis/directions-for-isolation/). See recipes at end of text.

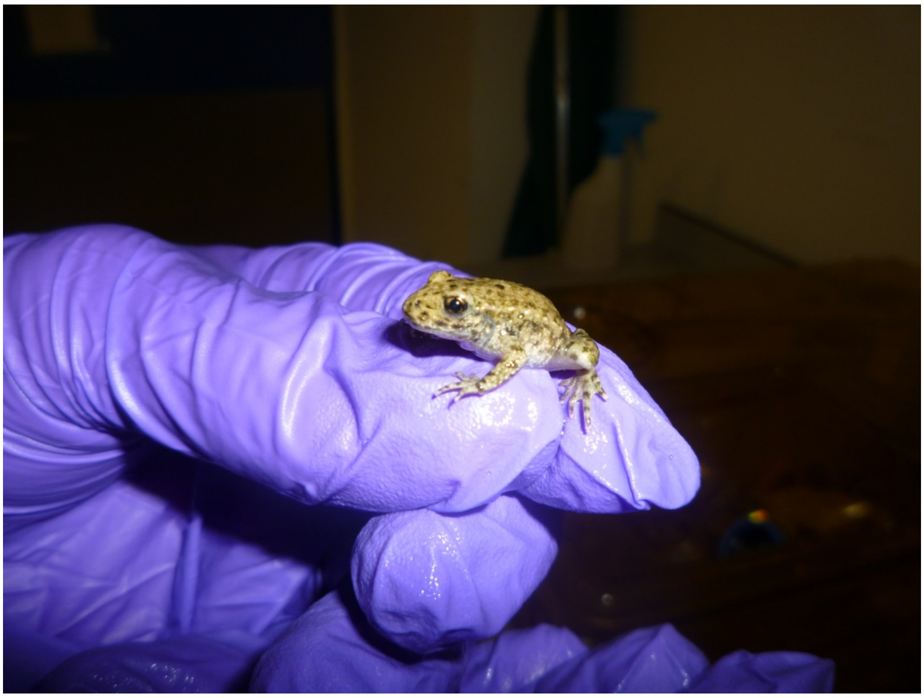

3. With fine scissors, clip off **either** the terminal 1–2 mm of the phalanges of the 4th hind toe (counting from the inside to outside) **or** (in small animals) the entire toe. Swab animals (supplementary protocol 2) and release at site of capture.

4. Change gloves or wash/disinfect hands between each animal.

5. If working in the field, place excised toe onto an agar plate for transport to laboratory or working site before moving onto step 6. Toes from the same location and species may be put on the same plate.

6. Clean amputated toe thoroughly by dragging it through nutrient + antibiotic agar with a sterile needle/forceps, ensuring that the entire toe comes into contact with the medium. Move the toe up and down through the depth of the medium. Every few mm, take the needle/forceps away from the toe and attached skin and wipe through the agar. The purpose of wiping the toe through agar is to remove surface bacteria and fungi. Bearing this in mind, wipe toe and skin back and forth; imagine what you are trying to do even though you cannot see bacteria or fungal contaminants.
    a. Several toes can be cleaned per plate as long as separate regions of the plate are used, or if the skin over the toe and the attached toe webbing are cut into 0.5 mm pieces, one plate can be used to clean tissue from each toe.

7. Once clean, use the needle to place the toe in a clear sterile tube containing liquid medium and store in a cool, dry place if possible. As some chytrids may be susceptible to antibiotics, we also include some tubes containing media without antibiotics.

8. For each tube record:
    a. Amphibian species, if known
    b. Location (as specific as possible) and date
    c. Any additional notes (male/female/juvenile; any outward signs of illness etc).

9. If in the field, keep tubes as close to 4 °C as is possible. If no refrigeration is available containers can be placed in shady streams or other cool places.

10. Check tubes every few days and discard any that turn cloudy.
    a. Visually prescreen tubes. Tubes that are not cloudy (yeast or bacterial contamination) or have mycelial 'balls' around the toe (non-chytrid fungal contaminants) can then be decanted into sterile 12-well lidded plates, each well containing 1 ml amount of liquid medium with and without penicillin and streptomycin. Incubate plates at temperatures that do not exceed 26 °C, ideally at 23 °C or below. Alternatively, decant onto nutrient antibiotic agar plates, allow liquid to dry, seal plate with Parafilm® and incubate as above.

    b. If in liquid medium, observe with an inverted compound microscope at 40, 100 and 400 X. If on agar medium, invert on a compound microscope and observe at 100X. Look for walled, spherical bodies (10–30 µm diam). Some of the bodies (sporangia) may have cleaved zoospores, which will look like 5–20 spherical bodies within the sporangia. In liquid medium chytrid sporangia adhere to the plate bottom and rhizoids are normally observed. In either liquid medium or on agar medium, motile *Batrachochytrium* zoospores (4–5 µm diam) may be evident around cleaned, infected skin within 1–2 days, but sometimes not for several weeks (for instance *B. salamandrivorans* takes several weeks). After the chytrid is growing well, aseptically transfer a 500 µL–1 mL aliquot to a sterile, screw-topped container containing 25–75 mL of liquid medium. *Batrachochytrium* grows best in *groups*, so do not separate sporangia from each other during transfers. Incubate for 1 or 2 weeks at 17–23 °C; after ample growth can be seen, store for up to 3 months at 4°C.

<u>Media recipes</u>

1% tryptone agar
- 10g tryptone
- 10g agar
- 1 L distilled water

1% tryptone liquid medium
- 10 g tryptone

- 1 L distilled water

mTGhL agar

- 8g tryptone
- 2g gelatin hydrolysate
- 4g lactose
- 10g agar
- 1 L distilled water

mTGhL liquid medium

- 8g tryptone
- 2g gelatin hydrolysate
- 4g lactose
- 10g agar
- 1 L distilled water

Adding antibiotics

•Autoclave media; remove from autoclave and place in a laminar flow hood or other clean area.

•Set timer for ~20 minutes.

• Flame spatula and cool briefly. With flamed spatula weigh out 0.2 g (200 mg/L) penicillin-G and 0.2-0.4 g (200-400 mg/L) streptomycin sulfate onto new, creased, glassine weigh paper

• Fold glassine paper and add antibiotic powder to liquid nutrient agar or broth that has cooled for at least 20 minutes.

note It is good practice to dissolve the antibiotics into 50 ml dH_2O in a falcon tube, then filter in the antibiotic mix through a 0.2 micrometer millipore filter

• Swirl to mix antibiotics

• If preparing nutrient agar, pour medium into 9 mm, plastic, culture plates in a laminar flow hood, or in quiet, clean area. Fill plates approximately 2/3 full; one liter of medium will make 40–46 plates.

• Replace lids on poured plates and leave in the laminar flow hood or in clean room for several hours or overnight before placing filled plates into plastic bags in which the plates arrived. Label each plate with a code for the type of medium, e.g., 1% T for 1 % tryptone and 1% T+ for 1% tryptone plus antibiotics
