# Supplementary material for: Development and worldwide use of non-lethal, and minimal population-level impact, protocols for the isolation of amphibian chytrid fungi
Source: Sci Rep. 2018 May 17;8:7772. doi: 10.1038/s41598-018-24472-2 (PMC5958081; doi:10.1038/s41598-018-24472-2)
Supplement: Supplementary file 2 — Swabbing protocol [file 41598_2018_24472_MOESM2_ESM.pdf]

***RACE Bd* and *Bsal* Swabbing Protocol**

First make sure that you have all the local permits and permissions from ethical committees to undertake the following protocol

Manuscript: Development and worldwide use of non-lethal, and minimal population-level impact, protocols for the isolation of amphibian chytrid fungi

Matthew C. Fisher, Pria Ghosh, Jennifer M. G. Shelton, Kieran Bates, Lola Brookes, Claudia Wierzbicki, Gonçalo M. Rosa, Rhys A. Farrer, David M. Aanensen, Mario Alvarado-Rybak, Arnaud Bataille, Lee Berger, Susanne Böll, Jaime Bosch, Frances C. Clare, Elodie Courtois, Angelica Crottini, Andrew A. Cunningham, Thomas M. Doherty-Bone, Fikirte Gebresenbet, David J. Gower, Jacob Höglund, Timothy Y. James, Thomas S. Jenkinson, Tiffany A. Kosch, Carolina Lambertini, Anssi Laurila, Chun-Fu Lin, Adeline Loyau, An Martel, Sara Meurling, Claude Miaud, Pete Minting, Serge Ndriantsoa, Frank Pasmans, Tsanta Rakotonanahary, Falitiana C. E. Rabemananjara, Luisa P. Ribeiro, Dirk S. Schmeller, Benedikt R. Schmidt, Lee Skerratt, Freya Smith, Claudio Soto-Azat, Giulia Tessa, Luís Felipe Toledo, Andrés Valenzuela-Sánchez, Ruhan Verster, Judit Vörös, Bruce Waldman, Rebecca J. Webb, Che Weldon, Emma Wombwell, Kelly R. Zamudio, Joyce E. Longcore, Trenton W. J. Garner

- Use tubed sterile dry swabs with a fine tip (if possible). We use the ones linked below
    - http://www.mwe.co.uk/microbiology-lab-supplies/dry-swabs-cotton-swabs-rayon-swabs-dacron-swabs/tubed-sterile-dryswabfine-tip-mw100/
- Ideally, use gloves when handling amphibians and change gloves in between each animal. Alternatively thoroughly wash/disinfect hands in between each animal however this will not prevent cross-contamination of samples with DNA.

1. Hold the animal firmly by its back legs. For caudatans, hold the animal on either side of its abdomen.

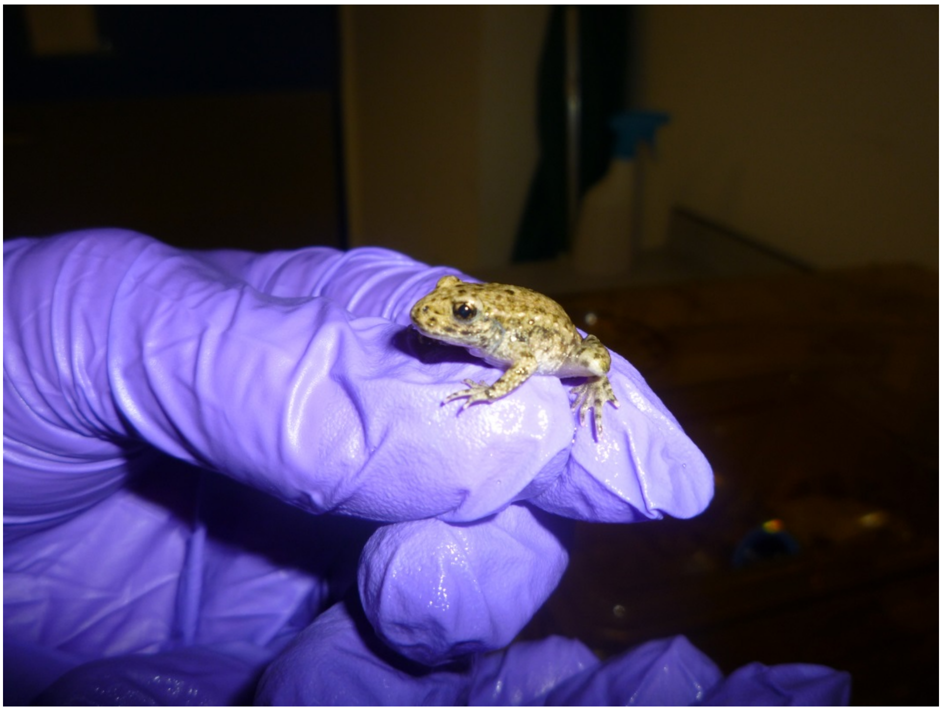

matthew.fisher@imperial.ac.uk

2. Firmly run the swab over the back and legs ten times. You will need to press quite hard on the skin as you swab, as you are trying to remove any fungal zoospores or sporangia embedded on the amphibian's skin. Rotate the swab as you swab to ensure it has all come into contact with the skin.

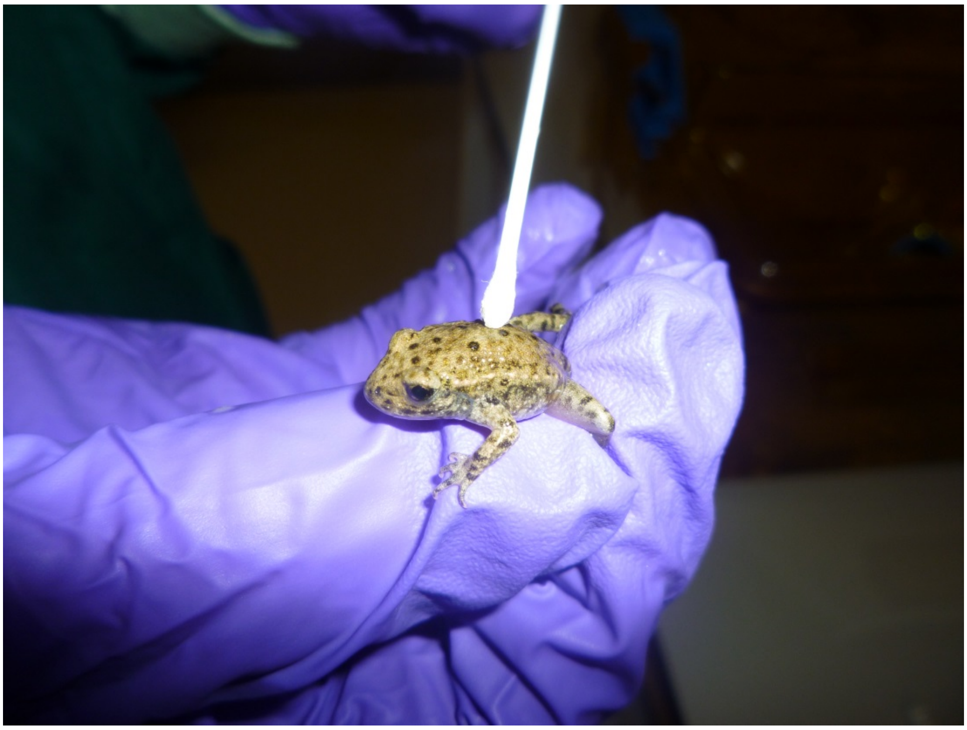

3. Next, swab the back legs and toes ten times per limb.

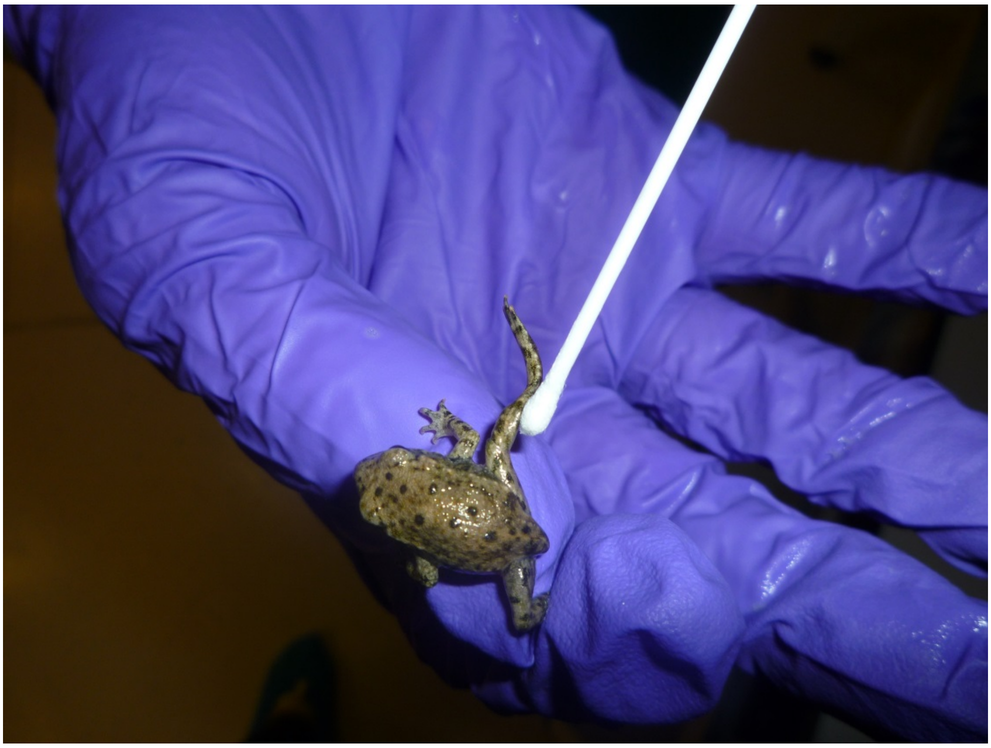

4. Flip the animal over to swab the pelvic patch and insides of the back legs ten times.

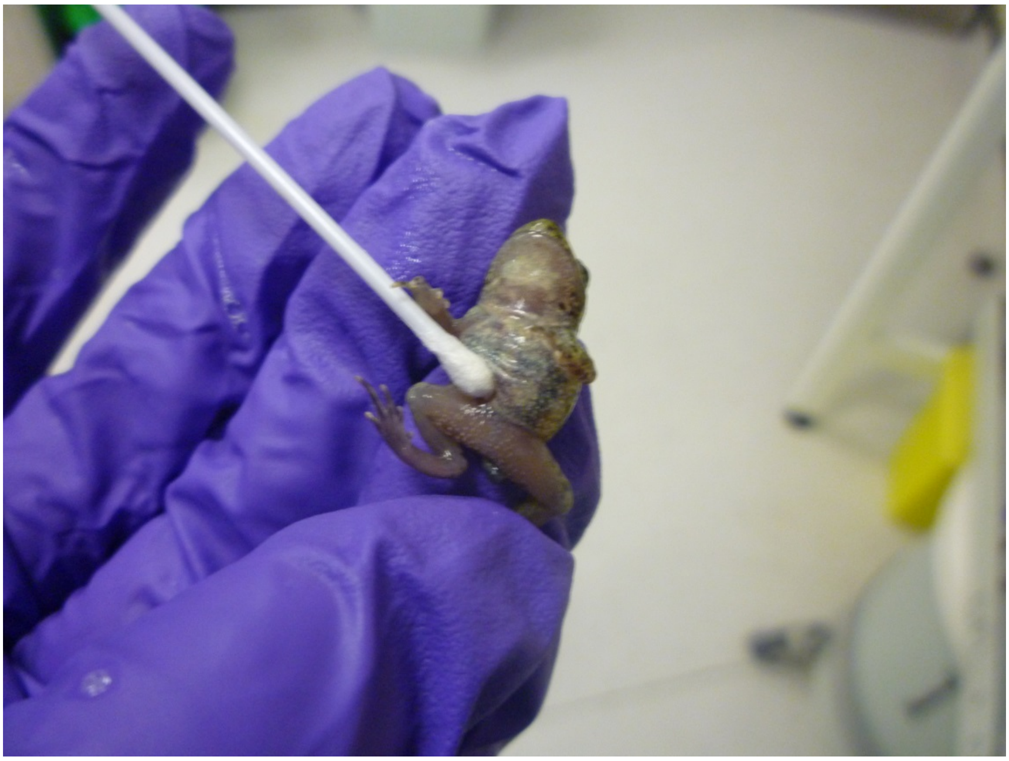

5. Pay particular attention to swabbing the back legs, toes and pelvic patch, because this is where *Bd* is most likely to be found.
6. Release the animal and store the swab at four degrees. Ambient temperature is not a problem for a few hours.
7. Give the swab a clear number either in a permanent marker or pencil
8. In a separate data file record for each swab:
    a. Location – as specific as possible
    b. Species (if known)
    c. Collector's/swabber's name
    d. Date
    e. Any additional information that may be relevant (e.g. male/female/juvenile; if the animal looked ill)
